# Sequential screening for depression in humanitarian emergencies: a validation study of the Patient Health Questionnaire among Syrian refugees

**DOI:** 10.1186/s12991-020-0259-x

**Published:** 2020-02-03

**Authors:** Danielle N. Poole, Shirley Liao, Elysia Larson, Bethany Hedt-Gauthier, Nathaniel A. Raymond, Till Bärnighausen, Mary C. Smith Fawzi

**Affiliations:** 1 Department of Global Health and Population, Harvard T.H. Chan School of Public Health, 665 Huntington Avenue, Boston, MA 02115 USA; 2 Harvard Humanitarian Initiative, Harvard T.H. Chan School of Public Health, 14 Story Street, Cambridge, MA 02138 USA; 3 Neukom Institute for Computational Science, Dartmouth College, Hanover, NH 03755 USA; 4 Department of Biostatistics, Harvard T.H. Chan School of Public Health, 665 Huntington Avenue, Boston, MA 02115 USA; 5 Department of Global Health and Social Medicine, Harvard Medical School, 641 Huntington Avenue, Boston, MA 02115 USA; 6 Jackson Institute of Global Affairs, Yale University, 55 Hillhouse Avenue, New Haven, CT 06520 USA; 7 Institute for Public Health, Faculty of Medicine, Heidelberg University, Im Neuenheimer Feld 130.3, 69120 Heidelberg, Germany; 8 Africa Health Research Institute, Mtubatuba, 3935 KwaZulu-Natal South Africa

**Keywords:** Mass screening, Validation studies, Psychometric, Mental health, Disaster, Armed conflict, Refugees

## Abstract

**Background:**

Despite the need for mental health surveillance in humanitarian emergencies, there is a lack of validated instruments. This study evaluated a sequential screening process for major depressive disorder (MDD) using the two- and eight-item Patient Health Questionnaires (PHQ-2 and PHQ-8, respectively).

**Methods:**

This study analyzed data collected during a cross-sectional survey in a Syrian refugee camp in Greece (*n* = 135). The response rate for each instrument was assessed, and response burden was calculated as the number of items completed. The sequential screening process was simulated to replicate the MDD classifications captured if the PHQ-2 was used to narrow the population receiving the full PHQ-8 assessment. All respondents were screened using the PHQ-2. Only respondents scoring ≥ 2 are considered at risk for symptoms of MDD and complete the remaining six items. The positive and negative percent agreement of this sequential screening process were evaluated.

**Results:**

The PHQ-2, PHQ-2/8 sequential screening process, and PHQ-8 were completed by 91%, 87%, and 84% of respondents, respectively. The sequential screening process had a positive percent agreement of 89% and a negative percent agreement of 100%, and eliminated the need to complete the full PHQ-8 scale for 34 (25%) respondents.

**Conclusions:**

The benefits of the sequential screening approach for the classification of MDD presented here are twofold: preserving classification accuracy relative to the PHQ-2 alone while reducing the response burden of the PHQ-8. This sequential screening approach is a pragmatic strategy for streamlining MDD surveillance in humanitarian emergencies.

## Background

Worldwide, 69 million people were forcibly displaced in 2018 [1]. The mental health needs of populations displaced by humanitarian emergencies are a significant yet often overlooked public health problem [2], and are exacerbated by post-migration stressors [3].

The high prevalence of mental health disorders, such as depression, among forcibly displaced populations frequently overwhelms local health system capacity, necessitating the integration of mental health services into the humanitarian response. International guidelines, including those of the Inter-Agency Standing Committee (IASC) [4] and Sphere [5], provide recommendations regarding the provision of mental health services to forcibly displaced populations. Additionally, the World Health Organization (WHO) has launched the comprehensive mental health Gap Action Programme Humanitarian Intervention Guide (mhGAP-HIG) to address the lack of mental health services in humanitarian emergencies, calling for consistent screening and task-shifting to non-specialist health care providers [6]. Efficacious psychological therapies for depression have been evaluated for humanitarian emergencies in low- and middle-income countries [7]; however, forced migrants experience inequitable access to health services relative to the general population [8, 9].

Despite consensus regarding the prioritization of population-level mental health [6, 10], there is no consistent approach to mental health surveillance in humanitarian emergencies. Underdiagnosis for mental health disorders remains higher among forcibly displaced populations compared to the general population [11]. The majority of studies of mental health in humanitarian emergencies include data from instruments with limited or untested validity and reliability among displaced populations [12]. In the absence of locally and culturally validated scales, applying robust standard measures as the first step for screening in emergency contexts may be necessary to expedite the rapid identification of individuals that need services [13]. Evaluations of mental health instruments for epidemiologic surveillance among populations displaced by humanitarian emergencies are therefore urgently needed [14].

As the first step in screening, instruments capturing self-reported symptoms reduce the cost and time requirements of formal clinical diagnostic interviews [15]. The desirability of shorter screening procedures is threefold—first, brief screening instruments reduce the participant response burden; second, shorter instruments may be more readily used in resource-constrained settings because of administration and cost efficiencies; and third, shorter instruments may result in fewer missing responses and thus reductions in the risk of invalidation due to missingness [16]. Overall, ideal screening instruments in humanitarian crises are (1) self-reported or administered by trained non-medical health workers [2], and (2) responsiveness to change [17], with (3) demonstrated acceptable response rate, reliability, and validity in displaced populations [14], (4) and a minimal response burden [16].

The Patient Health Questionnaire (PHQ) diagnostic algorithms incorporate DSM-V depression diagnostic criteria in brief self-report scales that produce estimates of base rates for depressive disorders and are sensitive to change in disorder status over time [18]. Additionally, the PHQ algorithms differentiate major depressive disorder (MDD) from mild and moderate depressive disorder, an important threshold for clinical diagnostic assessments and treatment. Moreover, consistent with the assessment timeframe recommended by the WHO, the questions evaluate symptoms over the last  two weeks [6].

Two approaches to MDD classification using PHQ algorithms have been widely used and validated: the PHQ-8 and PHQ-2. The eight-item version of the PHQ has been used extensively to screen for MDD in epidemiologic research [19, 20], including as an outcome for low-intensity intervention trials [21]. The PHQ-2 is a subset of the PHQ-8 that was developed for use in high-volume settings. The PHQ-8 and the PHQ-2 have both been widely validated in general clinical practice [22] and against a reference interview [23] with good sensitivity and specificity for MDD [17, 19]. Moreover, agreement between the PHQ-8 and PHQ-2 in detecting probable MDD has been demonstrated in a sample of pregnant women in the United States [24].

With the goal of minimizing the response burden while preserving screening validity, we evaluate a third approach: using empirical data, we simulate an MDD classification algorithm whereby individuals are first screened by the PHQ-2 assessment. Individuals flagged as having symptoms consistent with MDD by the PHQ-2 then go on to receive the full PHQ-8 assessment. The sequential screening approach may improve screening efficiency by reducing the number of items administered and managing the number of false-positive cases requiring follow-up [25]. The efficiency of sequential screening for mental disorders has been demonstrated in a primary care setting [25] as well as among refugee populations [26], and specifically using the PHQ-2 followed by the PHQ-8 among post-partum women [27] and an Arabic-speaking primary care population [28].

The objective of this study is to compare the performance of the PHQ-2 and the simulated PHQ-2/8 sequential screening process to classify symptoms consistent with MDD among a sample of Syrian refugees in Greece.

## Methods

### Data sources

The detailed methods of this study have been previously reported [29]. Briefly, this is an analysis of data collected during a face-to-face cross-sectional survey in a camp designated for Syrian refugees located in the Attica region of Greece in 2017. A mixed sampling procedure, consisting of two phases, was used to enroll a representative sample of 135 participants who were fluent in Arabic. In the first phase of sampling—designed to build trust—camp management announced that a research study was being undertaken on the topic of migrant health and adults were invited to volunteer to participate. Then, all eligible adults from half the housing units were recruited such that an even geographic distribution of the camp population was sampled. A standardized survey, including mental health measures and sociodemographic and displacement characteristics, was administered via a face-to-face interview by a member of the research team paired with Arabic–English interpreters. The survey was translated to and back-translated from Arabic prior to the interview. Participants that reported depressive symptoms in the last  two weeks were referred for assessment by an on-site psychologist.

### Major depressive disorder screening

The PHQ-8 is used as a reference standard for MDD classification in this study [19]. The PHQ-8 omits the ninth item of the PHQ-9 assessing suicidal ideation, but has been established to have similar validity in large-scale validation studies [19]. In the most recent validation study of the PHQ-9 in Arabic, the suicidal ideation item was the only item that increased instrument reliability if deleted [30].

The presence of depressive symptoms over the last  two weeks was evaluated by calculating severity scores for each item. On a four-point Likert scale ranging from “not at all” to “nearly every day,” respondents were asked to rate the degree to which each symptom applied to them over the last  two weeks. Items are scored from 0 (not present at all) to 3 (present nearly every day) and with a summary score ranging from 0 to 24. A cut-off score of ≥ 10 is used to classify the presence of MDD; this cut-off score was selected based on the findings of the Arabic validation study of the PHQ-9 [31] and evidence that identical scoring thresholds for depression severity may be used for the PHQ-9 and PHQ-8 [19].

Two approaches to minimize the response burden were simulated: the PHQ-2 and the PHQ-2/8 sequential screening process. The PHQ-2 consists of the first two items of the PHQ-8 and was developed for use in high-volume settings, such as humanitarian emergencies [17]. The PHQ-2 has the same response format as the PHQ-8, with summary scores ranging from 0 to 6 [17]. A previous validation study of the PHQ-2 in Arabic used the cut-off score of ≥ 3 based on the initial validation study [28]. Consistent with the recommendation that screening instrument thresholds be adjusted according to program objectives and capacity for reappraisal of all positive results [13], we examined possible cut-off scores of ≥ 2 and ≥ 3 in order to optimize the positive and negative percent agreement (PPA and NPA, respectively) relative to the PHQ-8 classification using the maximal Youden index for MDD [18, 20, 32].

### Sequential screening process

Major depressive disorder screening using the PHQ-2/8 sequential process was simulated with empirical data. The sequential screening process is as follows: (1) Responses to the PHQ-2 are evaluated for all participants. Participants who score below thresholds identified in previous validation studies in the general population in Arabic [28] and English [17] exit the screening process and are classified as “unaffected.” Participants who score above the threshold are considered at risk for symptoms consistent with MDD. (2) Responses to the remaining six items are scored for participants determined to be at risk for symptoms consistent with MDD by the PHQ-2, and are classified according to the PHQ-8 threshold standards.

### Statistical analysis

Descriptive statistics were calculated to summarize the psychometric properties. Floor or ceiling effects were defined as > 95% endorsement or < 5% endorsement, respectively. Reliability was measured as internal consistency captured within the PHQ-8 and PHQ-2 with Cronbach’s alpha.

Item response rate was described as the proportion of responses completed per item. Overall response rate was assessed as the proportion of respondents who completed all items in the instrument. Response burden refers to the strain placed on the respondent, often defined by factors such as the cognitive load, response fatigue, the format and mode of administration of the instrument, and the length of the instrument [33]. In this analysis, response burden is operationalized as the number of responses required to complete the screening method.

Respondents for whom four or more (≥ 50%) responses were missing were excluded from further analyses. Multivariate imputation by chained equations, in which missing values are imputed based on an individual’s observed outcome values and degree of similarity to demographic data observed in other participants, was performed for remaining missing values under the missing at random assumption [34].

#### Validity

This study analyzed aspects of validity relevant to comparisons of subscales with the full instrument, i.e., convergent and concurrent validity, in three ways. First, convergent validity was measured by assessing the likelihood ratio between the PHQ-2 summary score and the binary PHQ-8 classification. Second, concurrent validity, or the degrees to which the nominal MDD classifications obtained by the PHQ-2 and PHQ-2/8 sequential screening process were consistent with the PHQ-8 MDD classifications, was evaluated using Cohen’s kappa [35]. Finally, concurrent validity was assessed as the power of the two items in the PHQ-2 to predict the PHQ-8 classification using the deviance goodness of fit test. The deviance goodness of fit test accounts for the conditional dependence between the MDD classifications produced by the PHQ-2 and PHQ-8 as follows: a saturated logistic model in which the PHQ-8 score predicted the binary PHQ-8 MDD classification is built; then, the fit of a logistic model for the binary PHQ-8 MDD classification predicted by the PHQ-2 summary score is compared against the saturated model with the deviance goodness of fit test. By comparing the residual deviance against the *χ*^2^ distribution, the deviance goodness of fit test evaluates the fitted model against the saturated model [36]. The deviance test null hypothesis is that the PHQ-2 summary score adequately predicts MDD classification while the alternative hypothesis is that the model lacks an essential predictor. The deviance test accounted for the imperfect nature of the reference test and the assumed conditional dependence between the PHQ-2 and the reference test results.

We assessed systematic error in MDD classification between the PHQ-2 and PHQ-2/8 sequential screening process and the PHQ-8 with McNemar’s test. To compare the PHQ-2 and PHQ-2/8 sequential screening process, we evaluated their discriminate validity relative to the PHQ-8 MDD classifications. To this end, we calculated validity indices including the PPA and NPA, as recommended over sensitivity and specificity for comparisons with imperfect reference standards [37]. The PPA and NPA were used to calculate theoretical positive- and negative-predictive values (PPV and NPV, respectively) and estimates of the area under the curve (AUC) for ROC analysis of each screening method. Finally, the PPV and NPV were calculated for representative prevalence levels of (a) 5%, the level of depression worldwide [38]; (b) 35%, the prevalence reported in a metanalysis of depression among refugees [39], and (c) 81%, the highest reported depression prevalence among refugees [40].

#### Sensitivity analyses

We compared the characteristics of individuals who had missing responses to the sample characteristics to evaluate the potential for biased estimates. We used bivariate analyses (*χ*^2^ and Kruskal–Wallis tests) to compare these groups on gender, age, marital status, education, total time spent in displacement, and time spent seeking asylum in Greece.

Statistical analyses were conducted in Stata SE (v15·1) [41]. The reporting of our findings is consistent with the recommendations of the Guidelines for Reporting Reliability and Agreement Studies [42].

## Results

This analysis includes data from a total sample of 135 participants, representing 40% of the adult population residing in the refugee camp at the time of the survey. The mean age of the participants was 30 years (18–61 years); women comprised 41% of the sample; 74% of the participants had ever married; 67% had children; and 33% of participants had not attended secondary school, including 11% who had never attended school.

Item and instrument descriptive statistics and reliability indices are presented in Table 1. No floor or ceiling effects were detected. The most common symptom endorsed was related to feeling tired (*p4*, 75%), followed by feeling down or depressed, changes in sleep, and changes in appetite (items *p2*, *p3*, and *p5*, respectively, 72% each). The internal consistency of the items was low for the PHQ-2 (*α* = 0.45) and acceptable for the PHQ-8 (*α* = 0.78).

**Table 1:** Item and instrument descriptive statistics and reliability indices (*n* = 135)

Item	Mean	SD	Cronbach’s α*	Response rate (%)
Had little interest or enjoyment in doing things? (p1)	1.29	1.13	0.78	94
Felt down, depressed, or hopeless? (p2)	1.29	1.13	0.74	94
Had trouble falling or staying asleep, or sleeping too much? (p3)	1.55	1.22	0.75	97
Felt tired or having little energy? (p4)	1.35	1.10	0.73	95
Had a poor appetite or overeating? (p5)	1.45	1.18	0.76	97
Felt bad about yourself—or that you have let yourself or someone you cared about down? (p6)	1.02	1.18	0.77	93
Had trouble concentrating on things, such as reading or listening to music? (p7)	0.88	1.11	0.76	96
Moved more slowly, or the opposite, more quickly than usual? (p8)	1.00	1.22	0.74	93
PHQ-2	2.59	1.81	0.45	91
PHQ-8	9.91	6.00	0.78	84
Sequential screening	–	–	–	87

*SD* standard deviation, *PHQ-2* 2-item Patient Health Questionnaire, *PHQ-8* 8-item Patient Health Questionnaire

*The Cronbach’s alpha values corresponding to each item are the values resulting from the removal of the item from the full instrument

Overall, 91%, 87%, and 84% of respondents completed the entirety of the PHQ-2, sequential screening process, and PHQ-8, respectively. Items related to feeling bad about oneself and changes in movement had the highest proportion of missing responses (items *p6* and *p8*, 7% each). Six observations were missing four or more items and were excluded from the following analyses. A total of 114 respondents had no missing responses, while missing responses were interpolated for 15 respondents.

PHQ-2 cut-off points of ≥ 2 and ≥ 3 had Youden indices of 0.39 and 0.43, respectively, while the optimal empirical cut-off was calculated to be 2.5. A threshold of ≥ 2 for the PHQ-2 and sequential screening simulation was used to maximize sensitivity.

**Fig. 1:**
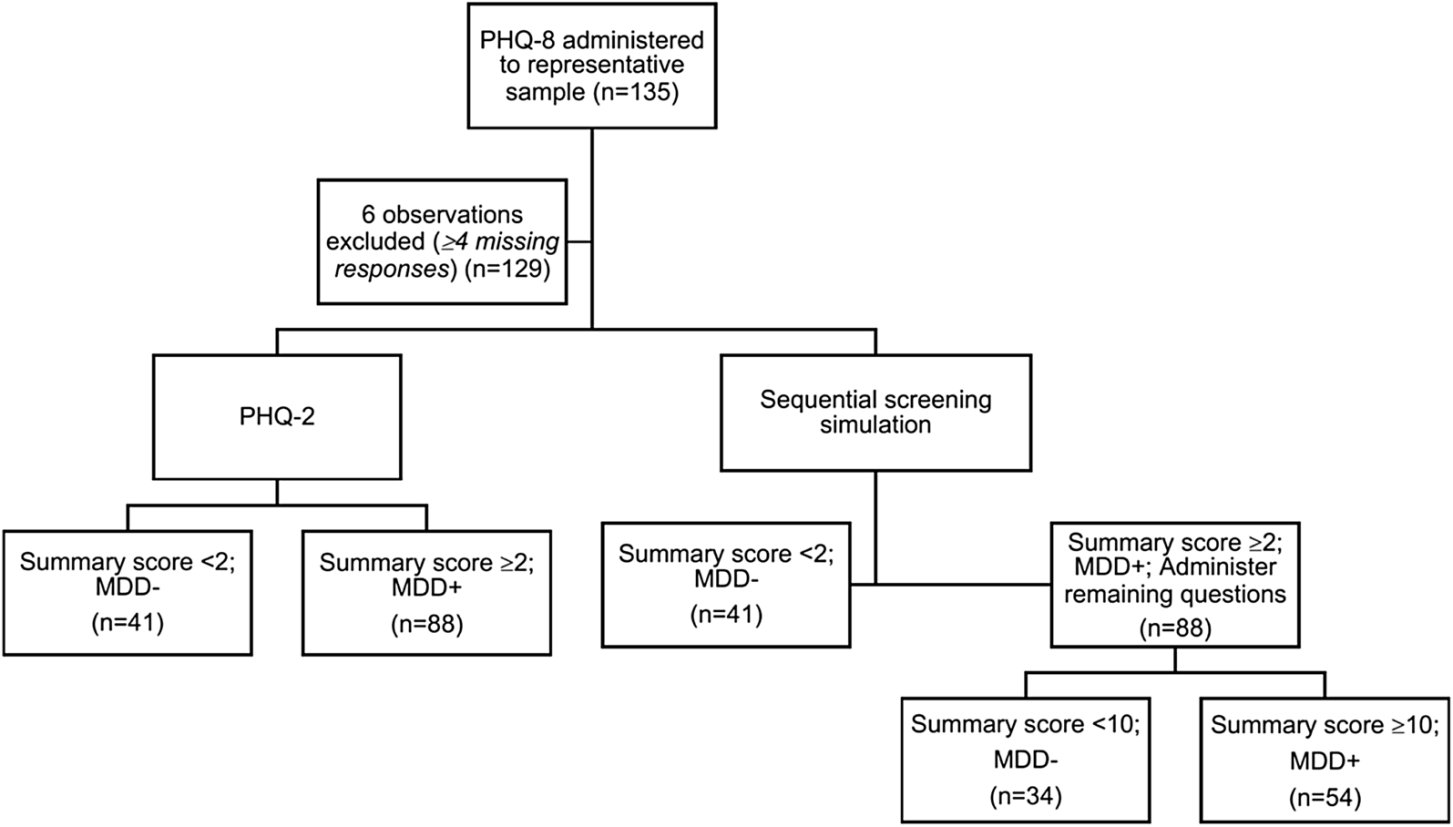
Simulated screening measures

A schematic of the simulated screening measures is presented in Fig. 1.

A total of 129 respondents were included in the comparative validity analyses. Nearly half of respondents were classified with MDD using the PHQ-8 (47%), 68% using the PHQ-2, and 42% using sequential screening. Two-by-two tables are presented in Additional file 1: Table S1. The response burden consisted of a total of 258 and 786 items for the PHQ-2 and sequential simulation, respectively, compared to 1032 for the PHQ-8.

### Instrument validity

The performance indices of the PHQ-2 and sequential screening methods against the PHQ-8 classifications are presented in Table 2. Cohen’s kappa values of 0.42 and 0.70 demonstrated weak agreement between the PHQ-2 and PHQ-8 and moderate agreement between the PHQ-2/8 sequential screening simulation and the PHQ-8, respectively, using recognized thresholds for agreement. Overall percent agreement with the PHQ-8 classification was 68% and 95% for the PHQ-2 and the PHQ-2/8 sequential screening simulation, respectively.

**Table 2:** Validity of the screening methods relative to the PHQ-8 MDD classification (*n* = 129)

Screening method	Major depressive disorder^a^			Systematic error^‡^ *p*-value	Cohen’s kappa (95% CI)	Validity indices^b^					
	+	−	Total			Overall agreement % (95% CI)	Positive agreement % (95% CI)	Negative agreement % (95% CI)	AUC	PPV (%)	NPV (%)
PHQ-2				0.000	0.42 (0.26–0.57)	68 (59–76)	89 (78–96)	50 (38–62)	0.69	61	83
Positive	54	34	88								
Negative	7	34	41								
Total	61	68	129								
PHQ-2/8 sequential screening				0.008	0.70 (0.55–0.80)	95 (89–98)	89 (78–96)	100 (95–100)	0.94	100	91
Positive	54	0	54								
Negative	7	68	75								
Total	61	68	129								

*PHQ-2* 2-item Patient Health Questionnaire, *95% CI* 95% confidence interval, *AUC* area under the curve for receiver operating characteristic analysis, *PPV* positive-predictive value, *NPV* negative-predictive value

^‡^McNemar’s test

^a^Respondents were defined as having major depressive disorder if they had a positive result on the PHQ-8

^b^Positive and negative percent agreement (PPA and NPA, respectively) were calculated as proxy measures of sensitivity and specificity, consistent with recommendations for comparisons with imperfect reference standards [37]

Classifications from both the PHQ-2 and the PHQ-2/8 sequential screening simulation were highly correlated with the binary PHQ-8 classification (PHQ-2, *p* < 0.001; the PHQ-2/8 sequential screening simulation, *p* < 0.001). The deviance test identified that the PHQ-2 was not missing a predictor relative to the PHQ-8 (*p* = 0.40).

There is evidence of a systematic difference in MDD classifications between the PHQ-8 and PHQ-2 instruments (McNemar’s test: *p* = 0.87). There is no evidence of a systematic difference in MDD classifications between the PHQ-8 and the PHQ-2/8 sequential screening instruments (McNemar’s test: *p* < 0.001).

The PHQ-2 classified MDD with 89% positive agreement and 50% negative agreement, a PPV of 61%, an NPV of 83%, and an AUC of 0.69. The PHQ-2/8 sequential screening simulation had a PPA of 89%, PPV of 100%, NPV of 91%, and AUC of 0.94. The observed NPA between the PHQ-2/8 and the PHQ-8 was 100%, an artifact of the study design.

The effects of different base rates of MDD on the classification accuracy of the PHQ-2 and sequential screening simulation using the calculated positive and negative percent agreement values as proxies for sensitivity and specificity are presented in Table 3.

**Table 3:** Effects of different base rates on MDD classification accuracy

Screening method	Representative prevalence^a^ (%)	PPV (%)	NPV (%)
PHQ-2	5	9	99
	35	49	89
	81	88	52
PHQ-2/8 sequential screening	5	100	99
	35	100	94
	81	100	68

*PHQ-2* 2-item Patient Health Questionnaire, *PPV* positive-predictive value, *NPV* negative-predictive value

^a^Baseline prevalence representative of (a) 5%, the level of depression worldwide [38]; (b) 35%, the prevalence reported in a metanalysis of depression among refugees [39], and (c) 81%, the highest reported depression prevalence among refugees [40]

Respondent characteristics associated with missing responses to the PHQ-2, sequential screening process, and PHQ-8 are presented in Additional file 1: Table S2. Trends were observed in the proportion of missing items across sociodemographic factors including gender, education, and marital status.

## Discussion

We report the first comparison of a brief and sequential screening method to reduce the response burden while preserving classification accuracy for MDD in a humanitarian emergency setting. Our study suggests that the sequential screening process for the detection of MDD could be a useful strategy for epidemiologic surveillance in humanitarian emergencies where mental health care is available. The sequential screening simulation detected 89% of respondents classified with MDD by the PHQ-8.

Due to the high volume of rapid assessments administered in response to humanitarian emergencies, response fatigue and subsequent low response rates are common. However, the response rate was ≥ 80% for all screening methods evaluated in this study, considered acceptable [43, 44]. The discrepancy in internal consistency of the items for the PHQ-2 (*α* = 0.45) and the PHQ-8 (*α* = 0.78) is in part expected due to the difference in the number of items. Nonetheless, the internal consistency of the PHQ-2 is considered unacceptable for an instrument of purportedly single dimensions.

The optimal threshold for the classification of MDD with the PHQ-2 was found to be ≥ 2. This threshold score has advantages over a threshold score of ≥ 3 in that more respondents with MDD are detected: compared to the commonly used threshold of ≥ 3, the threshold of ≥ 2 had superior PPA (89% compared to 69%, respectively) and NPA (100% for both). At a threshold score of ≥ 2, 68% of respondents would continue to complete the full PHQ-8 and 11% of MDD-positive respondents would be misclassified; at a threshold score of ≥ 3 or more, 47% would continue to complete the full PHQ-8 and 31% of MDD-positive respondents would be misclassified. The aim of the shorter screening methods (the PHQ-2 and sequential screening process) is to maximize detection of respondents with MDD while minimizing the response burden. Thus, we find that the threshold score of ≥ 2 has clinical advantages over a threshold score of ≥ 3 in that more respondents with MDD will be detected. This threshold was identified as optimal in previous validation studies of the PHQ-2 in primary care settings in New Zealand [23] and Australia [22].

Overall, 68%, 42%, and 47% of respondents were classified with MDD using the PHQ-2, the sequential screening simulation, and the PHQ-8, respectively. The difference in the proportion of respondents classified with MDD by the PHQ-8 and PHQ-2 was approximately 20%, with evidence of a systematic difference, in contrast to a previous finding that the instruments are equivocal in a Saudi primary care setting [28]. The difference in the proportion of respondents classified with MDD by the PHQ-8 and sequential screening simulation was smaller (5%), yet there was also evidence of a systematic difference between these proportions.

Concurrent validity between the screening instruments was established in several ways: first, agreement between the PHQ-2 and PHQ-8 was established by the deviance test (*p* = 0.001), accounting for the conditional dependence of the screening results. The sequential screening process had superior agreement with the PHQ-8 classification.

The PHQ-2 was equally sensitive to the positive detection of MDD compared to the sequential screening process (89% positive agreement for both). However, the trade-off for fewer items overall was a low negative percent agreement of 50%, resulting in 34 false-positive classifications. The sequential screening simulation, in comparison, had 100% negative agreement, with no false-positive classifications. Perfect negative agreement between the sequential screening simulation and PHQ-8 classification is an artifact of the sequential screening design, in that a respondent classified as “unaffected” by the PHQ-8 who nonetheless has a score ≥ 2 in the first step of the sequential screening process will ultimately be classified “unaffected”. However, we expect consistency in the classifications of a respondent completing the PHQ-8 after scoring ≥ 2 in the first step of the sequential screening process. Overall, 41 (32%) respondents were misclassified using the PHQ-2 alone, compared to 5% by the sequential screening simulation. While the PHQ-2 was highly sensitive, the large number of false-positive classifications may represent a barrier to implementation in resource-constrained settings. The value of a sequential screening process for MDD, whereby a two-question screen that is positive for depression is followed by the remaining items of the PHQ-8, has been previously demonstrated in a sample of post-partum women [27]. Our results extend the use of the sequential screening process to a humanitarian emergency.

The simulation of the sequential screening process demonstrates efficiencies similar to those described in previous screen-confirm strategies for mental health disorders in the general population [15] as well as among refugees [13]. The number of respondents requiring administration of the full PHQ-8 was reduced by 75% by the PHQ-2 and 24% by the sequential screening process. The sequential screening process was also found to reduce the response burden of screening for MDD, with only 68% of respondents progressing to the PHQ-8. While the reduction in the response burden achieved using the sequential screening process is modest, this reduction may nonetheless be valuable in settings with extreme resource constraints and high assessment burdens, such as humanitarian emergencies.

In addition to simulation with empirical data, we calculated the PPV and NPV with representative prevalence levels of depression. Screening instruments typically have relatively high false-positive rates (60–70%) in settings with a lower prevalence of depression (10%) [45]; however, we found the sequential screening simulation resulted in zero false-positive classifications. In contrast, positive results using the PHQ-2 alone should be confirmed by a full diagnostic given the high false-positive rate at all base rates of depression. In the context of high prevalence, a higher positive-predictive value (i.e., low false-positive classifications) minimizes unnecessary clinical intervention [46]. Both the PPV and NPV of the sequential screening simulation were robust across different base rates, suggesting this screening method represents a feasible and valid trade-off for surveillance.

This study is novel in the use of the deviance test to account for dependency in the comparison of the PHQ-2 and PHQ-8 classifications, a limitation of previous comparisons of these measures analyzed with ordinary least squares regression. An additional strength of this study is the high response rate, reducing the potential for bias due to high attrition observed in other studies [13]. This study also has several limitations. First, criteria for the diagnosis of MDD based on the DSM-V was not used as validation of the PHQ-8 in this study, as the purpose of the study was to compare brief screening instruments and the cost of a clinical diagnostic standard was prohibitive. The comparative method limits reporting of validity to agreement indices. A future study with a three-way comparison between the sequential screening method, the PHQ-8, and a clinical diagnosis would be useful for the calculation of unbiased sensitivity and specificity estimates. Second, the operationalization of response burden as a simple sum of items completed for each screening method could be improved by qualitative research examining in-depth information on the acceptability of the instruments. Third, further testing of the instruments in additional humanitarian contexts would advance our understanding of instrument performance and generalizability.

Recent advances in automating screening with technologies such as mobile phones may facilitate the use of sequential screening in such settings. Evidence that instrument performance is similar regardless of the mode of administration (e.g., patient self-report, interviewer-administered either in-person or by telephone) for self-reported depression measures [47, 48] supports the adoption of adaptive screening processes. Such automated procedures can result in the administration of fewer items without introducing error and thus measurement imprecision.

## Conclusions

The benefits of the sequential screening approach for the classification of MDD presented here are twofold: the preservation of classification accuracy relative to the PHQ-8 with a reduced response burden. These results suggest that the sequential screening approach is a pragmatic strategy to streamline MDD surveillance in humanitarian emergencies by reducing the response burden and facilitating the detection of MDD in settings with a scarcity of mental health specialists.

## Supplementary information

**Additional file 1: Table S1. **Two-by-two table (n = 129)**. Table S2.** Missingness by respondent characteristics (n = 135).

## Data Availability

The datasets generated and analyzed during the current study are not publicly available to protect the privacy of the participants, but are available from the corresponding author on reasonable request.

## Abbreviations

DSM-V: Diagnostic and Statistical Manual of Mental Disorders—Fifth Edition
IASC: Inter-Agency Standing Committee
MDD: Major depressive disorder
PHQ: Patient Health Questionnaire
mhGAP-HIG: Mental Health Gap Action Programme-Humanitarian Intervention Guide
WHO: World Health Organization
