# Supplementary material for: Sequential screening for depression in humanitarian emergencies: a validation study of the Patient Health Questionnaire among Syrian refugees
Source: Ann Gen Psychiatry. 2020 Feb 3;19:5. doi: 10.1186/s12991-020-0259-x (PMC6998825; doi:10.1186/s12991-020-0259-x)
Supplement: Supplementary file 1 — Additional file 1: Table S1. Two-by-two table (n = 129). Table S2. Missingness by respondent characteristics (n = 135). [file 12991_2020_259_MOESM1_ESM.docx]

**Table S1. Two-by-two table (n=129)**

| **Instrument and result** | **Major Depressive Disorder^a^** | | |
| --- | --- | --- | --- |
|  | **+** | **-** | **Total** |
| ***PHQ-2 ≥2*** |  |  |  |
| Positive | 54 | 34 | 88 |
| Negative | 7 | 34 | 41 |
| Total | 61 | 68 | 129 |
| ***PHQ-2 ≥3*** |  |  |  |
| Positive | 42 | 18 | 60 |
| Negative | 19 | 50 | 69 |
| Total | 61 | 68 | 129 |
| ***Sequential screening with PHQ-2 ≥2*** |  |  |  |
| Positive | 54 | 0 | 54 |
| Negative | 7 | 68 | 75 |
| Total | 61 | 68 | 129 |
| ***Sequential screening with PHQ-2 ≥3*** |  |  |  |
| Positive | 42 | 0 | 42 |
| Negative | 19 | 68 | 87 |
| Total | 61 | 68 | 129 |

PHQ-2 = 2-item Patient Health Questionnaire

^a^ Respondents were defined as having major depressive disorder if they had a positive result on the PHQ-8.

**Table S2. Missingness by respondent characteristics (n=135)**

|  |  |  | | **Respondents with missing items** | | | |  |
| --- | --- | --- | --- | --- | --- | --- | --- | --- |
| **Respondent characteristics** | **Total**  **(n=135)** | **PHQ-2**  **(n=12)** | **p-value** | | **Sequential screening (n=18)** | **p-value** | **PHQ-8**  **(n=21)** | **p-value** |
| Gender n (%) |  |  | 0·075* | |  | 0·026** |  | 0·007** |
| Men | 80 (59) | 10 (83) |  | | 15 (83) |  | 18 (86) |  |
| Women | 55 (41) | 2 (17) |  | | 3 (17) |  | 3 (14) |  |
| Age median (years, IQR) | 30 (24-37) | 34 (20-41) | 0·957 | | 29 (20-40) | 0·2587 | 30 (20-40) | 0·693 |
| Education n (%) |  |  | 0·004** | |  | 0·005** |  | 0·010** |
| None | 15 (11) | 0 (0) |  | | 0 (0) |  | 0 (0) |  |
| Any primary | 29 (22) | 1 (8) |  | | 2 (11) |  | 4 (19) |  |
| Any secondary | 38 (29) | 3 (25) |  | | 3 (17) |  | 3 (14) |  |
| Any tertiary or higher | 50 (38) | 6 (50) |  | | 11 (61) |  | 12 (57) |  |
| Prefer not to answer | 3 (2) | 2 (17) |  | | 2 (11) |  | 2 (10) |  |
| Marital status n (%) |  |  | >0·001** | |  | >0·001** |  | 0·002** |
| Single, never married | 34 (25) | 4 (33) |  | | 7 (39) |  | 7 (33) |  |
| Ever married | 99 (73) | 6 (50) |  | | 9 (50) |  | 12 (57) |  |
| Prefer not to answer | 2 (2) | 2 (17) |  | | 2 (11) |  | 2 (10) |  |
| Total time displaced (months) median (IQR) | 12 (11-36) | 9 (1-48) | 0·933 | | 1 (1-17) | 0·6036 | 1 (1-17) | 0·841 |
| Time in Greece (months) median (IQR) | 10 (10-11) | 10 (5-11) | 0·488 | | 10 (8-11) | 0·6970 | 10 (5-11) | 0·258 |

* χ2 test or Kruskal-Wallis test p<0·1.

** χ2 test or Kruskal-Wallis test p<0·05.
